# Expanding the phenotype of E318K (c.952G > A) MITF germline mutation carriers: case series and review of the literature

**DOI:** 10.1186/s13053-021-00189-8

**Published:** 2021-07-21

**Authors:** Leandro Jonata Carvalho Oliveira, Aline Bobato Lara Gongora, Fabiola Ambrosio Silveira Lima, Felipe Sales Nogueira Amorim Canedo, Carla Vanessa Quirino, Janina Pontes Pisani, Maria Isabel Achatz, Benedito Mauro Rossi

**Affiliations:** Serviço de Oncogenética – Centro de Oncologia Hospital Sírio-Libanês, Rua Dona Adma Jafet, 91, 01308-050 São Paulo, Brazil

**Keywords:** *MITF*, c.952G > A, p.E318K, Hereditary cancer, Multigene sequencing panel, Breast cancer

## Abstract

**Background:**

The microphthalmia-associated transcription factor gene (*MITF*) belongs to the *MYC* supergene family and plays an important role in melanocytes’ homeostasis. Individuals harboring *MITF* germline pathogenic variants are at increased risk of developing cancer, most notably melanoma and renal cell carcinoma.

**Case presentation:**

We describe a cohort of ten individuals who harbor the same *MITF* c.952G > A (p.Glu 318Lys), or p.E318K, germline pathogenic variant. Six carriers developed at least one malignancy (4 cases of breast cancer; 1 cervical cancer; 1 colon cancer; 1 melanoma; 1 ovarian/fallopian tube cancer). A significant phenotypic heterogeneity was found among these individuals and their relatives. Breast cancer was, overall, the most frequent malignancy observed in this case series, with 13 occurrences of 60 (21.67 %) total cancer cases described among the probands and their relatives.

**Conclusions:**

Our retrospective analysis data raise the hypothesis of a possible association of the *MITF* p.E318K pathogenic variant with an increased risk of breast cancer.

## Introduction

The microphthalmia-associated transcription factor gene (*MITF*) plays an important role in melanocytes’ homeostasis [1]. It is one of the helix-loop-helix leucine-zipper transcription factors that are part of the *MYC* supergene family [2]. There are nine different isoforms of *MITF (*-A, -J, -C, -MC, -E, -H, -D, -B and -M), each one with a 5’ exon specificity. Nevertheless, these isoforms have exons 2 to exon 9 in common, which encode the transcription factors` functional domains, that include the transactivation domain and the basic helix-loop-helix leucine-zipper [3]. The bHLH-Zip is formed by three regions: the basic motif, the helix-loop-helix motif and the leucine-zipper motif. The latter two are important for the interaction of the melanocyte inducing transcription factors with each other or with other proteins with a similar structure. As a result, a homodimer and/or heterodimer with transcription factor’s functions is generated [4]. The basic motif binds to some specific areas of the DNA, in order to facilitate the dimers to regulate genes’ activity. There are other functional domains other than the bHLH-Zip, which play a role in post-transcriptional activity, through ubiquitination, phosphorylation or other processes [5].

The M-isoform is most expressed in the melanocyte lineage and it activates transcription of genes involved in melanogenesis and pigmentation, as well as other genes that are crucial for cells’ differentiation, survival, invasiveness and metabolism [4]. The A-isoform is expressed in the kidney. However, its exact role in renal cells transformation has not been quite well defined [2]. *MITF* is also involved in development of other cell types, such as osteoclasts and mast cells. *MITF* loss-of-function mutations cause inherited disorders in neural crest development, Tietz and type 2a Waardenburg syndromes, characterized by pigmentation abnormalities and other alterations. Germline *MITF* mutations have been described to increase the risk of melanoma and, more recently, kidney cancer [1].

Development of cancer is a multifactorial process. Tumoral cells can own a capacity of resisting cell death, evading growth suppressors, sustaining proliferative signaling, inducing angiogenesis, replicative immortality and activating invasion and metastasis. Genomic instability is one of the processes that lead to these hallmarks [6]. Germline pathogenic variants may be a component which initiates this genome instability and, hence, cancer formation [7].

The emergence of next-generation sequencing (NGS) panels for inherited cancer has enabled analysis of a wide variety of genes. Historically, germline testing for a limited number of genes was offered for patients who met phenotypic criteria for a hereditary cancer syndrome. Currently, with spreading of NGS panels, the same phenotype can be associated with different pathogenic germline variants. Moreover, a phenotypic heterogeneity in patients carrying the same germline mutation has also been observed [8, 9].

We hereby describe the characteristics of ten patients who harbor a *MITF* germline pathogenic variant. These cases illustrate the phenotypic heterogeneity among patients carrying the same germline mutation.

## Methods

All patients included were evaluated at the Oncogenetics Unit at Hospital Sírio-Libanês, located in São Paulo, Brazil. A retrospective analysis was conducted at the Registry of Hereditary Cancer Syndromes at Hospital Sirio-Libanes for individuals harboring *MITF* pathogenic variants identified in a multigene panel of 84 genes (Invitae® multi-cancer panel). Confirmation of the pathogenic variants identified in the NGS followed Invitae®’s routine, which performs other assays, such as Sanger sequencing, Pacific Biosciences SMRT sequencing, MLPA, MLPA-seq or Array CGH, for all clinically significant observations, except for individually validated variants and variants previously confirmed in a first-degree relative focused in only a select subset of patients [10, 11].

Clinical data from medical charts were available and each patient’s familial history of cancer was collected. Descriptive analyses of patients’ pedigree charts were performed to extract genotype-phenotype correlations. Odds Ratio for pathogenic *MITF* allele frequency was calculated with an online calculator (https://www.medcalc.org/calc/odds_ratio.php). Median allele frequency of *MITF* in our findings was compared to the MAF in an Online Archive of Brazilian Mutations (http://abraom.ib.usp.br), which contains genomic variants obtained with whole-exome and whole-genome sequencing from SABE, a census-based sample of elderly individuals from São Paulo, Brazil’s largest city (http://abraom.ib.usp.br) [12]. Patient data are presented in an aggregated or non-identifiable manner and no informed consent was deemed necessary by the institutional ethics committee board.

## Results

During the period comprised between the first and last test positive for a pathogenic variant in MITF (07/22/2016 to 12/19/2019), a total of 1056 multigene NGS panels were performed in our institution, of which ten had identified a pathogenic variant in MITF (0.9 %). Here we describe these ten unrelated probands that carried the germline *MITF* c.952G > A (p. Glu318Lys) pathogenic variant. All probands were submitted to testing with a multigene panel of 84 genes (Invitae® multi-cancer panel). Confirmation assays were performed according to Invitae®’s criteria. This multi-cancer panel offers only the analysis of the c.952G > A (p.Glu318Lys) variant in MITF, according to their national guidelines. Hence, no other pathogenic variants in MITF were searched in our cohort. The Odds Ratio for pathogenic *MITF* allele frequency in our population compared to general Brazilian population was 22.4 (95 % CI 11.93 to 42.05, *p* < 0.0001). We do recognize that this increased OR may be due to our highly selected population (i.e., patients evaluated in an Oncogenetics Unit), compared to the general Brazilian population.

Among the ten selected probands, no additional pathogenic or likely pathogenic variants were found in any other analyzed genes, except for the fumarate hydratase (FH) gene alteration described below. Variants of uncertain significance were not described due to their currently unknown clinical relevance. Six of the probands had a history of cancer (Table 1). Indications for testing varied greatly among the analyzed patients. The six aforementioned individuals with a history of malignancy were referred to genetic counselling due to a combination of personal and familial history. The four remaining probands were referred solely due to a positive family history for malignancies, as described below.

**Table 1:** Summary of personal and family history of cancer in *MITF* E318K germline mutation families

Cases	Phenotype	Family history
M, 44y	Asymptomatic	Skin BCC FDR Ovary 42y FDR
F, 59y	Breast 57y Cervical 30y	Skin CBC 58y FDR Prostate 59y FDR Colon 75y FDR
F, 50y	Breast 49y	Colon 54y FDR Prostate 79y FDR
F, 50y	Breast 57y	Breast 62y SDR
F, 66y	Colon 62y	Stomach 54y FDR Stomach 62y FDR Stomach 65y FDR Stomach 45y SDR Colon 52y SDR
F, 60y	Breast 49y	Breast 58y FDR Bilateral Breast 45y FDR Pancreas 75y SDR Prostate 75y SDR Stomach 65y SDR
F, 65y	Asymptomatic	Prostate 50y FDR Prostate 50y FDR Ovary 52y SDR
F, 56y	Ovary/tube 52y Melanoma 55y	Esophagus 48y FDR Stomach 67y FDR Bilateral Breast 68y SDR
F, 36y	Phyllodes Benign 36y	Bilateral Breast 48y FDR Pancreas 55y SDR
M, 44y	Asymptomatic	Breast 67y FDR Intrahepatic ducts 65y FDR Prostate 68y SDR Prostate 74y SDR Throat 78y SDR

Legends: *F* Female; *M* Male; *BCC* Basal cell carcinoma; *FDR* First degree relatives; *SDR *Second degree relatives

A total of 54 malignancies were reported among 337 relatives registered in our database. A review of the probands’ family histories revealed that 19 of their 74 (25.67 %) first-degree relatives (FDR), as well as 27 of 186 (14.52 %) second-degree relatives (SDR) and 8 of 77 (10.39 %) of third-degree relatives (TDR), had at least one diagnosed malignancy throughout their lives (Table 2).

**Table 2:** Number of patients with cancer among probands that carried *MITF* p.E318K variant and their relatives, compared to the total of relatives mentioned on familial history

	Probands	FDR	Paternal SDR	Maternal SDR	Paternal TDR	Maternal TDR
**Cancer history**	6 (60 %)	19 (25 %)	10 (8 %)	17 (24 %)	3 (14 %)	5 (8,9 %)
**Total**	10	74	116	70	21	56

*FDR* First-degree relatives, *SDR* second-degree relatives, *TDR* third-degree relatives

Among the probands with a positive history of cancer, four (4/8) females developed breast cancer between ages 49 and 57. One of these individuals also had a history of cervical cancer at age 30. One proband had a history of both ovary/fallopian tube cancer and melanoma after age 52, and another had a history of colon cancer. Among the four other probands, one had a history of a benign phyllodes tumor. The other three were asymptomatic and had no known history of neoplasms or malignancies (Table 3). Screening for melanoma with annual dermatoscopy was recommended for all probands regardless of previous history of cancer. Screening for kidney tumors was not routinely recommended due to lack of evidence to support its benefit. The possibility was discussed with the probands and regular ultrasounds for kidney assessment were performed based on patient’s choice.

**Table 3:** Cancer history among probands that carried *MITF* p.E318K variant and their relatives

Cancer type	Proband	FDR	Paternal SDR	Maternal SDR	Paternal TDR	Maternal TDR
Breast	4	4	2	1	1	1
Prostate	-	4	2	2	-	-
Colon	1	2	2	5	-	3
Stomach	-	4	1	1	-	-
Skin (non-melanoma)	-	2	2	-	-	-
Melanoma	1	-	-	-	-	-
Pancreas	-	-	-	3	-	-
Esophagus	-	1	1	-	1	-
Ovary	-	2	-	-	-	-
Oviduct	1	-	-	-	-	-
Cervix	1	-	-	-	-	-
Testis	-	-	1	-	1	-
Bladder	-	-	1	-	-	-
Lung	-	-	1	-	-	-
Head and Neck	-	-	-	1	-	1
Leukemia	-	-	-	1	-	-
NOS	-	-	1	3	-	-

*FDR* First-degree relatives, *SDR* second-degree relatives, *TDR* third-degree relatives, *NOS* Not otherwise specified

Among the probands’ FDR, breast cancer was the most frequently observed malignancy, with four individuals with a positive history, two of them with bilateral breast cancers. Prostate and gastric cancers were also observed in four cases each in this group. Colon cancer was the most frequent malignancy among SDR, with seven cases described, followed by prostate cancer, with four cases, and breast cancer, with two cases of unilateral disease and one case of bilateral disease. Three occurrences of pancreatic cancer were described in SDR. Colon and breast cancers were also the most frequent cancer types diagnosed in TDR, with three and two cases respectively. Cascade testing for family members was recommended in all cases but definite results are not available at the moment.

Overall, breast cancer was the most frequently observed malignancy among probands and their relatives, accounting for 13 (considering bilateral cases as single occurrences) out of 60 (21.67 %) of total cancer cases. Analyzing individual family histories separately (Table 1), heterogeneous patterns of cancer incidence with diverse histologic subtypes arose:

- Proband 1 is an asymptomatic male with no personal history of cancer. His sister had ovary cancer at age 42 and his brother had a history of basal cell carcinoma (BCC) of the skin. Three of his maternal aunts, as well as one maternal cousin, had colon cancer.
- Proband 2 is a female with a history of breast and cervical cancers. Her father had a history of prostate cancer at an early age and her mother had a diagnosis of colon cancer after age 75. One of her paternal uncles had a history of multiple malignancies (bladder, testicular, colon and prostate cancer), and her maternal grandfather was diagnosed with leukemia at an advanced age.
- Proband 3 is a female diagnosed with breast cancer under the age of 50. A brother, two of her mother’s siblings and a maternal female cousin all had a positive history for colon cancer. Her father had prostate cancer and a maternal aunt had a history of breast cancer.
- Proband 4 is also a female presenting with breast cancer, with a maternal aunt with a history of breast cancer.
- Proband 5 is a female who had colon cancer at age 62. Her paternal grandmother, her father and two of her siblings had gastric cancer, and one of her nephews had colon cancer.
- Proband 6 is a female with a history of breast cancer and two sisters also with breast cancer, one of them with bilateral malignancies. Her mother’s family has a history of prostate, pancreatic, gastric and other non-specified cancers.
- Proband 7 is an asymptomatic female. Two of her brothers were diagnosed with prostate cancer, both of them at age 50, and her mother had a history of ovarian cancer.
- Proband 8 is a female who had an ovarian/fallopian tube carcinoma and a cutaneous melanoma diagnosed almost three years apart. Her father had gastric cancer and her father’s family has a history of multiple cases of breast cancer, including a paternal aunt and her daughter with bilateral and unilateral breast malignancies respectively.
- Proband 9 is an asymptomatic female whose mother had synchronous bilateral breast cancer under age 50. Her mother’s family had a positive history for breast and colon cancer, and her maternal grandfather and one maternal uncle had a history of pancreatic cancer.
- Proband 10 is an asymptomatic male who also carries a *FH* likely pathogenic variant (c.1431_1433dup). Her mother had breast cancer and two maternal uncles and one paternal uncle have positive histories for malignancies, one of the maternal uncles with a history of head and neck cancer associated with tobacco use, and the two others diagnosed with prostate cancer after age 70.

## Discussion

The *MITF* (microphthalmia-associated transcription factor) gene encodes a protein called melanocyte inducing transcription factor. This protein helps to control the development and function of melanocytes, retinal pigment epithelial cells (specialized cells in the eye), osteoclasts and mast cells. In the melanocytes, its dimers bind to genes that control the production of the melanin pigment. Melanocytes are also found in the inner ear and play an important role in hearing. The retinal pigment epithelial cells nourish the retina, which detects light and color [13]. *MITF* loss-of-function mutations have been associated with autosomal-dominant syndromes characterized by hypopigmentation and sensorineural hearing loss, such as Waardenburg Syndrome (WS) and Tietz Syndrome (TS) [1].

Mutations in the *MITF* gene can alter the helix-loop-helix or leucine-zipper motif or even result in an abnormally small version of the protein, which has been identified in people with Waardenburg syndrome type 2 A (WS2A). More than 35 mutations in *MITF* have been associated with WS2A. Mutations may disrupt the formation of dimers, impairing the development of melanocytes and resulting in a shortage of melanocytes in certain areas of the skin, hair, eyes, and inner ear [13]. There are four types of WS described (WS type 1, 2, 3 and 4), all with similar clinical features, including sensorineural hearing loss, hypopigmentation of skin and hair and pigmentary disturbances of the irises (hypoplastic blue irises and/or heterochromia). WS1 and WS3 are associated with mutations in *PAX3*, and WS4 has been shown to be due to mutations in the receptor–ligand pair *EDN3/EDNRB* or in the transcription factor *SOX10* [14].

The *MITF* mutations that cause Tietz syndrome either delete or change a single amino acid in the basic motif region of the melanocyte, inducing the transcription factor structure to create abnormal dimers. As a result, most of the dimers are unavailable to be transported into the cell nucleus and bind to DNA, affecting the development of melanocytes and the production of melanin. Tietz Syndrome is a fully penetrant autosomal-dominant inheritance characterized by profound congenital hearing loss and generalized hypopigmentation. Researchers suggest that Tietz syndrome may be a severe form of Waardenburg syndrome [13, 15].

*MITF* also plays an important role in melanoma. *MITF* gene amplification has been observed in 20-30 % of metastatic melanoma and is associated with decreased overall survival [16]. *MITF* mutations found in metastatic melanoma samples were able to bind DNA and activate expression of melanocyte-specific promoters; some showed increased potential to form colonies [13]. At the transcriptional level, *MITF* controls genes involved in cell survival (*BCL2, HIF1A, BCL2A1*), migration (*DIAPH1, MET*) and proliferation (*CDK2, TBX2, CDKN1B*), providing important signals for growth of melanoma cells [17]. Strub and colleagues showed that sustained inhibition of *MITF* induces a G0/G1 growth arrest on cells and their entrance into senescence, a program associated with cessation of the proliferation potential [18]. MITF in conjunction with mutant *BRAF*(V600E), an activating mutation commonly present in melanocytic lesions, trigger transformation of immortalized melanocytes, functioning as a melanoma oncogene. Reduction of *MITF* activity sensitizes melanoma cells to therapeutic agents. Hence, targeting *MITF* in combination with *BRAF* or cyclin-dependent kinase may offer a rational therapeutic way on melanoma [16].

Yokoyama and colleagues performed the whole-genome sequencing of probands from several melanoma families in order to identify genes associated with familial melanoma. They identified one individual carrying a germline missense substitution p.E318K (c.952G > A, NM000248.3) in the melanoma-lineage-specific oncogene *MITF*. The co-segregated variant with melanoma indicated a possible intermediate-risk variant (log of odds score of 2.7 under a dominant model). Consistent with this, the variant was found to double the risk of melanoma for carriers in a large Australian population-based case-control study. The same effect was observed in a case-control study in the United Kingdom [19]. Moreover, the variant was found with a similar allele frequency in a group of Italian melanoma patients [20] and in another Australian study [21]. These studies show that the *MITF* E318K variant is enriched in those with multiple primary melanomas or a family history of melanomas, suggesting that the mutation in *MITF* predisposes to familial and sporadic melanoma. This mutation is very rare in the general population, with an allele frequency raging from 0.003 in the French population to 0.0085 in the UK population [20]. In the Brazilian population, a study evaluating the prevalence of Germline *TERT* and *MITF* mutations in Brazilian Melanoma-Prone Patients showed an allele frequency of 0.004 in control group (1 out of 125 healthy controls) and 0,01 in melanoma patients’ cohort [22]. An estimated 5-10 % of all cutaneous melanoma cases occur in patients with a strong family history of melanoma. About 45 % of these familial melanomas have been attributed to inheritance of a mutation in a highly penetrant predisposition gene: *CDKN2A* (cyclin-dependent kinase 2 A) and less frequently in *CDK4* (cyclin-dependent kinase 4), *BAP1* (breast cancer associated protein-1), *TERT* (telomerase reverse transcriptase), and *POT1* (protection of telomeres 1). The other cases of familial melanoma are likely due to inheritance of lower-penetrance predisposition genes such as *MC1R* (melanocortin 1 receptor) and *MITF* in combination with inheritance of polymorphisms. In addition, there may be shared environmental exposures, culminating in a familial pattern of melanoma inheritance. Melanoma also might emerge as a “subordinate cancer”, in the context of other cancer syndromes (Cowden syndrome, Li Fraumeni syndrome, breast and ovarian cancer syndrome), which carry a poorly defined, but elevated risk of this malignancy [23, 24].

The p.E318K mutation alters *MITF* SUMOylating, increasing the MITF transcriptional activity with upregulation of downstream genes. Two SUMOylating sites, one in the N-terminal region and the other in the C-terminal region, have been identified in the MITF sequence. Codon 318 is located in a small-ubiquitin-like modifier (SUMO) consensus YKXE site in the C-terminal part of *MITF*, and germline missense substitution in *MITF* (Mi-E318K) changes the glutamic acid at codon 318 into a lysin, severely impairing *MITF* SUMOylation. An analysis of genome wide occupancy reveals a global increase in Mi-E318K occupied loci, indicating that SUMOylation-deficient *MITF* E318K protein may result in the regulation of distinct sets of genes. Transcriptomic analysis indicates that the *MITF* E318K signature is related to cell growth, proliferation and inflammation [2].

Clinically, characteristics that have been associated with the p.E318K mutation in the oncogene *MITF* are high nevus counts, development of multiples primary melanomas, onset of melanoma before the age of 40, and non-blue eye color [25]. Although rare, other cancers have been associated with this variant. In a study performed by Ghiorzo et al. in an Italian population, an association with nodular melanomas in E318K-positive patients was observed. In this study, the E318K variant appeared to be involved in the development of pancreatic cancer in melanoma-prone families. This finding provided the first indication that *MITF* may be involved in the development of PC, in melanoma families [20].

A rare functional variant p.E318K in the *MITF* gene has also been implicated in renal cell carcinoma susceptibility. Mi-E318K enhanced *MITF* protein binds to the HIF1A promoter and increases its transcriptional activity, compared to wild-type MITF. HIF1A is a pathway targeted by kidney cancer susceptibility genes [26]. Bertolotto and colleagues showed that Mi-E318K occurred at a significantly higher frequency in patients affected with melanoma, renal cell carcinoma (RCC) or both cancers, than in controls. Mi-E318K carriers had an increased risk higher than fivefold of developing melanoma, RCC or both cancers. Therefore, they proposed that *MITF* might have a role in conferring a genetic predisposition to co-occurring melanoma and RCC. In melanoma and renal carcinoma cells, *MITF* E318K appears to enhance clonogenicity, migration and invasion, consistent with a gain-of-function role in tumorigenesis [2]. Interestingly, only one case of melanoma and no cases of renal cancer were found in our cohort, which may suggest that other risk factors should be taken into consideration when assessing the overall hereditary risk of these tumors in patients who carry the E318K variant. The association between this variant and melanoma, renal cancer, as well as other malignancies, may be related to shared environmental or polygenic risk factors, rather than this specific MITF polymorphism. In both contexts, familial history should be carefully evaluated.

In addition to the relationship of this variant with melanoma, RCC and pancreatic cancer, a study identified its association with pheochromocytoma/paraganglioma [27]. A recent meta-analysis of data from nine published studies regarding the relationship of personal history of melanoma and MITF E318K demonstrated that MITF E318K was significantly correlated with melanoma. A systematic review was also performed, evaluating the prevalence of the MITF(E318K) variant in multiple cancer cohorts by germline whole-exome sequencing data from the TCGA panel and from several genetically enriched cohorts. Among the 25 cancers tested (including RCC, PC, pheochromocytoma, paraganglioma and breast cancer), uterine carcinosarcoma (OR 9.24; 95 % CI 2.08–37.17; *p* = 0.024) and melanoma (OR 2.15; 95 % CI 1.03–4.37; *p* = 0.061) exhibited the strongest associations with the variant [28].

There is paucity of data related to germline changes in *MITF* and predisposition to breast cancer. A Polish population-based study examined the prevalence of the E318K and V320I MITF mutation germline in cancer patients and its association with the risk of cancers of different sites, such as melanoma or kidney, lung, prostate, colon or breast cancer. This study showed no statistically significant association between the variants and the tumors analyzed, including breast cancer. In the control population of this study, the E318K variant was detected in 0.19 % and the V320I variant was detected in 0.14 % [29]. In the systematic review and meta-analysis by Guhan et al., the risk of developing breast cancer in MGH genetically-enriched breast cancer with the MITF(E318K) mutation was increased, but significance was not reached (OR 2.67; 95 % CI 0.87–7.83; *p* = 0.111) [27]. Nevertheless, breast cancer was one of the most frequent malignancies in our probands, and also among their families.

Data regarding potential roles of MITF in oncogenic events and metabolic changes in breast cancer cells are indeed scant but some significant correlations have been demonstrated. In a Korean study designed to characterize the somatic mutation profiles in patients with TNBC, the description of homozygous deletion of *MITF*, *EPHA5* and *ACSL3* were significantly associated with an increased risk of recurrence or distant metastasis in patients treated with adjuvant chemotherapy [30]. In a more recent analysis, Osmanbeyoglu and colleagues evaluated the impact of the MITF transcription factor on gene expression in basal breast cancer cells. They found that MITF silencing frequently led to downregulation of pro-oncogenic factors, as c-Myc, c-Myc target genes, IL1B, NT5E (CD73) and molecules related to tumor immune evasion, as well as to consistent upregulation of genes associated with immune activation and cell adhesion, thus suggesting a tumor-promoting role for MITF activity in basal breast cancer [31]. Furthermore, as mentioned previously, the role of MITF in the regulation of the BCL2 gene activity has already been studied in melanoma cells. In that regard, Haq and colleagues demonstrated direct activation of BCL2A1 by MITF and significant reduction of BCL2A1 mRNA levels after MITF knockdown [32]. The increase of BCL2A1 activity is known to lead to suppression of apoptosis and appears to impact on cell survival and resistance to treatment in breast cancer [33, 34]. Therefore, it is possible that increased MITF activity may be implicated in survivability changes in breast cancer cells.

Another recent work of interest, a comprehensive review by Goding and Arnheiter, delineates multiple MITF regulation activities and interactions associated with cell cycle regulation and carcinogenesis. For instance, the authors suggest that MITF high activity may increase expression of *CDKN1A* and *CDKN2A* and induce a p21/p16-dependent cell cycle arrest [35]. They also consider MITF to be an up-regulator of CDK2 expression and a positive enhancer of cyclin genes *CCNB1* and *CCND1*. CDK2 activity and *CDKN2A* and *CCNB1* overexpression have been consistently linked to breast cancer cell development and survival [35, 36, 37, 38]. Moreover, an interesting observation made by Goding and Arnheiter regards the shared characteristics in function and mode of regulation of TFEB/TFE3 and nonmelanocyte isoforms of MITF and the inference that, since highly related, those factors may also share a large number of target genes [35]. Considering that TFEB has been implicated likewise in breast cancer development as an autophagy inducing factor and via other mechanisms[39, 40, 41], investigating these similarities between MITF and TFEB might lead to a different potential explanation for the role of mutated MITF in the scenario discussed in our case series. Evidence hence supports the notion that MITF might play an ancillary part in breast cancer oncogenesis via multiple interactions of its transcription factor. Consequently, a mutated MITF with high transcriptional activity might help to enable the crosstalk of breast cancer cells with their tumor microenvironment and to enhance their survival and growth. We hypothesize that the presence of the p.E318K MITF germline variant might lead to augmented expression of the aforementioned pro-oncogenic, and possibly antiapoptotic, genes, thus facilitating oncogenic events not only in melanoma but also in breast cancer cells and possibly other tumors.

Based only on the presence of their pathogenic p.E318K variant, the patients described in this study would not necessarily qualify for screening and follow-up for cancers other than melanoma, under current guidelines recommendations. It is important to notice, though, that with the exponential uptake of next-generation sequencing–based hereditary cancer panels, the analysis of large data sets has furthered our interpretation of well characterized hereditary cancer predisposition syndromes’ phenotypic spectra. In a study with 165,000 patients undergoing hereditary cancer predisposition testing, gene-specific associations were evaluated with six different phenotypes, as well as the performance of NCCN genetic testing criteria for BRCA-related breast and/or ovarian cancer syndrome and Lynch syndrome. Among patients who met testing criteria for only *BRCA1/2* or only Lynch syndrome and presented positive pathogenic variants (PV), mutations were present in the respective genes in 33.1 and 46.2 %. Conversely, 5.8 % of patients with PVs in *BRCA1/2* and 26.9 % of patients with PVs in Lynch syndrome genes did not meet respective testing criteria [8]. A prospective study compared the proportion of pathogenic germline variants detected by universal tumor–normal sequencing with the proportion that would have been detected based on clinical guidelines. Interestingly, 55.5 % of actionable germline findings would not have been detected in these cancer patients if only those who met established guidelines criteria were tested [42]. Care should be taken when facing a patient with no established guidelines criteria for specific syndromes, but with a phenotype that may suggest a germline pathogenic variant.

Our study has limitations. Our sample of 10 probands is small, and it does not allow for any definitive conclusion. Hence, it must be regarded as hypothesis generating. In addition, segregation analyses were not performed, since only a small number of relatives was tested for the MITF E318K pathogenic variant, due to access and cost issues. However, we believe our findings are significant and may lead to future studies that can expand the knowledge regarding the role of MITF in predisposition to different solid tumors and can aid in delineating specific guidelines to other scenarios, including breast cancer.

## Conclusions

Breast cancer was one of the most frequent malignancies in our probands and also among their families. Our retrospective analysis data raise the hypothesis of a possible association of the *MITF* p.E318K pathogenic variant with an increased risk of breast cancer. Additional studies are definitely needed to identify and report more breast cancer patients with E318K mutations, in order to clarify its role as a risk factor or pathogenic variant contributing to the development of breast cancer. Better estimates of the associated risk will improve the genetic counseling and screening recommendations.

## Data Availability

The data that support the findings of this study are available from the corresponding author, LJCO, upon reasonable request.
